# Mechanistic Model of Fatigue in Ultrasonic Assisted Machining

**DOI:** 10.3390/ma17194889

**Published:** 2024-10-05

**Authors:** Reza Teimouri, Marcin Grabowski

**Affiliations:** Faculty of Mechanical Engineering, Cracow University of Technology, 31-864 Cracow, Poland; marcin.grabowski@pk.edu.pl

**Keywords:** ultrasonic assisted machining, anti-fatigue design, surface integrity, mechanistic modeling, stress-based approach, regression analysis

## Abstract

Anti-fatigue design in the machining process of aviation material requires advanced processes to enhance the surface integrity and a holistic model which can optimize the process aiming at maximum fatigue life. In the present study, the axial ultrasonic assisted milling process was utilized to machine the Inconel 718 while the process executes the thermomechanical cutting and peening action simultaneously. To optimize the process factors, a hybrid model using a combination of regression analysis and an analytical model was developed to correlate the machining factors, i.e., vibration amplitude, cutting velocity and feed rate to fatigue life. Herein, the former was used to map the process inputs to surface integrity aspects (SIAs), viz. roughness, hardness and residual stress; then, the SIA was mapped to fatigue life through a stress-based approach. The obtained results revealed that there is close agreement between the measured and predicted values of fatigue life where the prediction error is less than two times the dispersion. On the other hand, applying ultrasonic vibration at the highest amplitude together with the maximum feed rate and cutting velocity yield significant improvement in fatigue life, i.e., three times the same condition without ultrasonic vibration in light of the enhancement of compressive residual stress and work hardening of the surface layers.

## 1. Introduction

Poor machinability of Inconel 718 limits the surface integrity through the formation of tensile residual stress on subsurface layers and rough surface quality. External field assisted machining can be utilized for enhancement of the surface integrity of machined Inconel 718, which will have great impact on its fatigue life. The association of ultrasonic vibration to metal cutting processes is a successful type of field assisted machining that was reported to have several advantages like less cutting force and longer tool life on steels, aluminums, titanium and other metallic alloys. However, the impact of ultrasonic vibration on the surface integrity and fatigue life of Inconel 718 as a difficult-to-machine material needs further studies [1].

In this context, Suarez et al. [2] studied the effect of ultrasonic assistance on the surface integrity-induced fatigue life of Inconel 718 in an ultrasonic assisted milling process. They showed that the surface roughness, hardness and fatigue life of machined material are enhanced in ultrasonic assisted machining (UAM) compared to conventional milling. In another attempt [3], they compared the surface integrity and induced fatigue life of Inconel 718 machined by different machining techniques, including conventional milling, ultrasonic assisted milling, abrasive waterjet machining and wire electrical discharge machining. It was found from the results that among the foresaid treatments, ultrasonic vibration-assisted milling results in less surface roughness, further hardness and bigger magnitude and depth of compressive residual stress, which result in a longer lifetime. Yin et al. [4] proposed the process of ultrasonic peening-milling to enhance the machined surface integrity of Inconel 718 in a high-speed cutting process. The obtained results reported significant enhancement of the roughness, hardness, compressive residual stress and, subsequently, fatigue life of the machined Inconel 718 by the proposed process compared to conventional high-speed milling. Therefore, it can be concluded that ultrasonic assisted cutting is a promising technique to be used as finish machining to enhance the durability of Inconel 718.

In the aviation industry, which uses Inconel 718 as a mainstream material, the durability and life of material can be considered a main machinability indicator. Thus, to maximize the fatigue life of products in the design stage subject to productivity and economical aspects, an accurate trustable model is required to predict the final fatigue life by adjusting the process factors. The design, based on experimental trials and errors, is costly and time consuming since fatigue examinations are very long specifically in terms of high-cycle fatigue life. This limits finding the effect of the main parameters due to high demands to experimental works. As a very valid example, in all the above reviewed studies, only a limited number of experiments were carried out to identify the fatigue life of Inconel 718 since lots of experimental resources are required to produce comprehensive research.

To address the foresaid problem, the development of a physics-based model validated by experiments provides the means to effectively understand the underlying fatigue failure mechanism and to optimize the process in terms of anti-fatigue machining. In spite of existing research which used physics-based models to analyze the fatigue life of materials after machining, all of them correlate fatigue to SIA [5], not directly to the process parameters. Therefore, the development of a fatigue life model which can be directly adjusted by process factors and that includes the impact of SIA opens a new research direction that merits further study.

**Figure 1:**
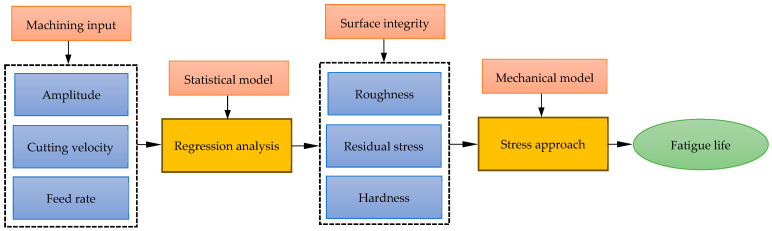
Scheme of investigation.

The present study takes the lead to directly correlate the life of the machined samples to process parameters through a mechanistic model where SIA (roughness, residual stress and hardness) are correlated to process parameters (vibration amplitude, cutting velocity and feed rate) using regression analysis. Then, the fatigue life is correlated to the statistically modeled SIA using a mechanical stress-based approach. Figure 1 shows the scheme of investigation.

## 2. Materials and Methods

### 2.1. Mechanistic Modeling

The term mechanistic modeling is a combination of mechanical and statistical models to correlate process factors to the fatigue lives of samples. Here, a statistical model is used on the basis of regression analysis including linear, two-factor and quadratic terms aiming at a correlation between process inputs, i.e., amplitude, cutting velocity- and feed rate-to-surface roughness indices (*Ra*, *Rz*, *Rt* and *Rsm*), mean values of residual stress (*σ^Rs^_Ave_*) and hardness (*H_Ave_*).

To find the response of process factors with respect to process quality characteristics, second-order mathematical models of machining force and surface roughness were developed through response surface methodology according to the following equations:

(1) $$Y=b_{0}+\sum_{i=1}^{k}b_{i}X_{iu}+\sum_{i=1}^{k}b_{ii}{X_{iu}}^{2}+\sum_{i=1}^{k}b_{ij}X_{iu}X_{ju}$$

where *Y* represents the foresaid SIAs; *b*_0_, *b_i_*, *b_ii_* and *b_ij_* are the coefficients; *X_iu_* is the variable (*A*, *Vc* and *fz*); *u* is the experiment number (1–18); *k* is the factor number (1–3); *X_iu_*^2^ is the higher order term of the variable; and *X_iu_* and *X_ju_* are the interaction terms.

The adequacy of the model is checked by analyses of variance through identifying significant terms and *R*^2^ examination. In the present study, the statistical modeling was implemented in the MINITAB 17 statistical package.

The mechanical term of this mechanistic modeling on the basis of a stress-based approach was used to correlate the abovesaid SIA to fatigue life.

In the present work, the stress-based approach was utilized to predict the lifetime of machined samples.

The stress-based approach was the earliest proposed approach for fatigue life prediction and is still the most frequently used. In this approach, the fatigue life (number of cycles *Nf*) is related to the applied stress range or the stress amplitude. As mentioned above, the surface integrity, including the geometrical, mechanical and metallurgical parameters, has been shown to have a significant effect on fatigue behavior.

Accordingly, the model relates the lifetime to applied fatigue load or stress amplitude using an exponential model that includes some coefficients. However, to include the role of SIA in the final lifetime, the stress-based approach can be modified. The general form of the model is written as follows [5]:

(2) $$\sigma_{eqv}^{k}(p)-\sigma_{l}^{k}(p)=\sigma_{f}{\left(N_{f}\right)}^{c}$$

where *σ_eqv_* is equivalent stress, *σ_l_* denotes the stress limit, ***p*** is the surface integrity parameter, *N_f_* denotes the number of cycles, *σ_f_* and *c* are the empirical parameters and *k* denotes the exponent of stress in the S-N curve formula.

In order to include the impacts of SIA on calculation of fatigue life, *σ_eqv_* can be defined as a function of surface roughness and residual stress; also, *σ_l_* can be dependent on hardness [5]. According to previous studies [6], the residual stress and roughness can impact the equivalent stress by adjusting the stress concentration factor (SCF). On the other hand, the hardness can impact the fatigue limit stress due to resisting the initiation and propagation of cracks. Based on the report by Xiong et al. [7], the equivalent stress is

(3) $$\left\{\begin{array}{c} \sigma_{eqv,max}=K_{t}\sigma_{max,nom}\\ \sigma_{eqv,min}=K_{t}\sigma_{min,nom} \end{array}\right.$$

where *σ_eqv_*_,*max*_ and *σ_eqv_*_,*min*_ denote maximum and minimum local stress at the surface of the sample; *σ_max_*_,*nom*_ is the maximum nominal stress that is applied during fatigue testing, and *K_t_* denotes SCF. The relationship between the stress concentration factor and roughness parameters was introduced by Arola [8] as

(4) $$K_{t}=1+n\sqrt{\lambda }\left(\frac{Ra}{\rho }\right)\left(\frac{Rt}{Rz}\right)\;$$

where *n* equals 1 for shear and 2 for tension, *ρ* is the root radius of the surface valley and *λ* is the ratio of the *Rsm* as surface unevenness pitch to *Rt* as maximum height to depth distance.

(5) $$\lambda =\frac{R_{sm}}{R_{t}}$$

Accordingly, the effective fatigue stress concentration factor *K_f_* is calculated using following equation:

(6) $$K_{f}=1+q\left(K_{t}-1\right)$$

where *q* in this work is set to 0.25 for a V-shaped notch with a circular root [9].

**Figure 2:**
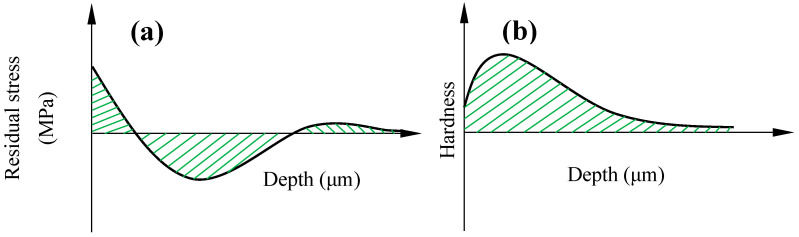
Distribution of (**a**) residual stress and (**b**) hardness over certain depth.

To include the impact of residual stress, it is assumed that it has uniform distribution up a certain depth of the workpiece. Accordingly, Equation (2) is modified as follows:

(7) $$\left\{\begin{array}{c} \sigma_{eqv,max}=K_{f}\sigma_{max,nom}+\sigma_{rs}\\ \sigma_{eqv,min}=K_{f}\sigma_{min,nom}+\sigma_{rs} \end{array}\right.$$

where *σ_rs_* is the mean value of residual stress distributed up to a depth where the compressive residual stress reaches zero, as shown in Figure 2a. Equation (6) can be rewritten by introducing the mean stress and stress amplitude using Smith’s method [10].

(8) $$\sigma_{ar}=\sqrt{\sigma_{max}\sigma_{a}}=\sqrt{\sigma_{a}\left(\sigma_{a}+\sigma_{m}\right)}$$

where *σ_m_* and *σ_a_* are the local mean stress and stress amplitude, which can be calculated using following equation:

(9) $$\left\{\begin{array}{c} \sigma_{m}=\frac{1}{2}K_{f}{(\sigma }_{max,nom}+{\sigma_{min,nom})+\sigma }_{rs}\\ \sigma_{a}=\frac{1}{2}K_{f}{(\sigma }_{max,nom}-\sigma_{min,nom}) \end{array}\right.$$

Mukarami et al. [11] reported that the fatigue strength limit of a material can be correlated to hardness by a linear relationship:

(10) $$\sigma_{lr}=\alpha H+\beta$$

where *α* and *β* are empirical coefficients, which is reported in the work carried out by Song et al. [5], and *H* is the mean hardness distribution (as shown Figure 2b) of the machined material.

Therefore, by underlying each term in Equation (1), it can be rewritten as follows:

(11) $$\sigma_{ar}\left(K_{f},\sigma_{rs}\right)-\sigma_{lr}(H)=\sigma_{f}{\left(N_{f}\right)}^{c}$$

In order to calculate the lifetime, the unknown terms in Equation (10), *K_f_*, which is a function of roughness, *σ_rs_* and *H* are calculated using the regression analysis, which includes the effect of process factors.

### 2.2. Experimental Work

**Figure 3:**
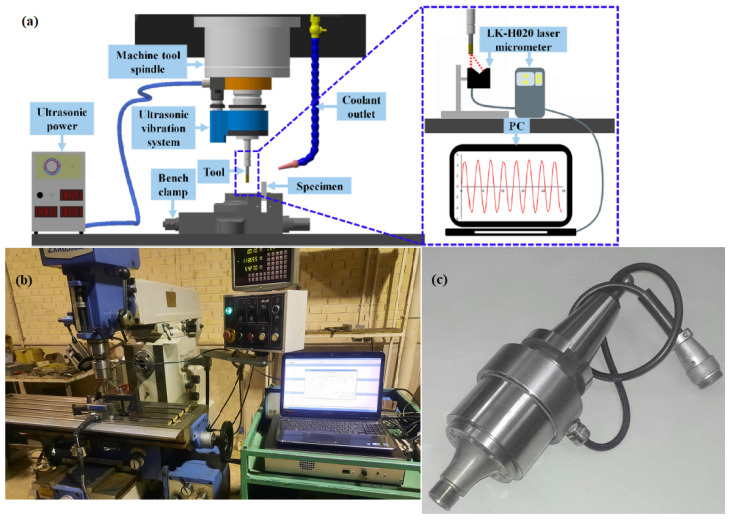
(**a**) Schematic of ultrasonic assisted milling setup [4]; (**b**) milling machine equipped with ultrasonic apparatus; (**c**) hand–made BT30 ultrasonic spindle.

A series of ultrasonic assisted milling experiments were carried out on an Inconel 718 sample with dimensions of 200 mm × 6 mm × 11 mm and the mechanical properties presented in Table 1. Before the main experiments, the samples were ground on both sides to achieve flatness and remove contamination remaining from previous treatments. The cutting tool which was utilized to mill the upper surface of the samples is a 10 mm, PVD TiAlN coated carbide tool manufactured by SECO Co. A setup like the one used in the work carried out by Yin et al. [4] was prepared to conduct experiments. Figure 3a shows a schematic diagram of the experimental setup. The milling machine used in the present work is a China-made mill center machine equipped with a hand-made rotary-vibratory ultrasonic apparatus BT-30 (Figure 3b,c) that is mounted on the head of the milling machine.

The surface roughness of the samples was measured using a MahrSurf surface profilometer (Mitutoyo, Japan) with a cut-off value of 0.08mm. The device is able to provide surface profiles with all the required characteristics mentioned in Section 2, i.e., *R_t_*, *Ra*, *Rz* and *Rsm*, using the obtained profile.

The residual stress of the samples was measured using an X-ray diffraction method with a XRD machine (StressTech, USA) with a *Cr-Kβ* radiation line (wavelength *λ* = 2.085 A). For Inconel 718, the diffraction plane {311} with a Bragg angle of 150.89° was employed during measurements. To measure the through thickness residual stress, layers of samples with thickness of 50 μm were removed through an electropolishing method using a solution of acetic acid (94%) and perchloric acid (6%). It should be emphasized that the mean residual stress distribution was calculated using the area under the residual stress depth distribution, as schematically shown in Figure 2a.

The through thickness microhardness of the samples was measured by a Vickers microhardness machine (Shimadzu, Japan) with an applied indentation load of 50 g under a 15 s dwell time. The hardness distribution was measured in each 50 μm depth direction. The microhardness was measured up to the depth where the hardness reached the hardness of the received material and the mean value of the hardness was calculated through the area under the curve of the hardness–depth, as shown in Figure 2b.

The fatigue life of the samples was measured using a four-point bending test as schematically shown in Figure 3c with a frequency of 10 Hz at a stress ratio of *R* = 0.1, with two loading conditions under maximum stress values of *σ_max_* = 750 MPa and *σ_max_* = 600 MPa. Since this type of fatigue test is somehow expensive and time consuming, six samples were subjected to fatigue testing to confirm the results derived from the developed hybrid model.

The experiments were carried out after identifying the important process factors affecting ultrasonic assisted milling processes, which are cutting speed, feed rate and ultrasonic vibration amplitude. The main experiments were planned based on the full factorial design considering two levels for vibration amplitude (0 and 8 μm), cutting velocity (25, 50 and 75 m/min) and feed per tooth (0.04, 0.06 and 0.08 mm). Accordingly, a total of 18 experiments were carried out and the SIAs were measured to develop regression models. Table 2 illustrates the experimental design and measured values of the SIA.

It needs to be pointed out the reasons for selection of the foresaid process range was because of equipment limitation and keeping the concept of high-speed machining in our work. The selection of cutting velocities less than 25 m/min does not meet the concept of high-speed machining; on the other hand, when the cutting velocity is higher than 75 m/min, the machine spindle experiences some vibrations which damage the stability of the process. Moreover, a selection of feed per tooth less than 0.04 mm conflicts with the concept of hard machining; also, if *f_z_* is bigger than 0.08 mm, the cutting force becomes higher, which may impact the effectiveness of our ultrasonic apparatus.

## 3. Results and Discussion

### 3.1. Statistical Modeling of SIA

A second-order polynomial regression analysis using constant, linear, two-factor interaction and quadratic terms was used to correlate the UAM process factors, i.e., amplitude, cutting velocity and feed rate, to surface roughness indices (*Ra*, *Rt*, *Rz* and *Rsm*), mean residual stress distribution (*σ^RS^*) and mean hardness (*H_ave_*). The corresponding mathematical equations excluding the insignificant equations are found in Equations (12)–(17):

(12) $$Ra\;\left(\mu m\right)=0.547-0.006A-0.001V_{c}+1.15f_{z}+0.1\times 10^{-4}V_{c}^{2}+35f_{z}^{2}-0.066Af_{z}-0.011V_{c}f_{z}\;$$

(13) $$Rz\;\left(\mu m\right)=1.91-0.021A-0.0036V_{c}+4.03f_{z}+0.4\times 10^{-4}V_{c}^{2}+122.5f_{z}^{2}-0.23Af_{z}-0.038V_{c}f_{z}$$

(14) $$Rt\;\left(\mu m\right)=2.46-0.027A-0.0047V_{c}+5.18f_{z}+5\times 10^{-4}V_{c}^{2}+157.5f_{z}^{2}-0.3Af_{z}-0.049V_{c}f_{z}$$

(15) $$Rsm\;\left(\mu m\right)=27.93-0.123A-0.3237V_{c}+595f_{z}+0.3\times 10^{-2}V_{c}^{2}+2900f_{z}^{2}-4.71Af_{z}$$

(16) $$H_{ave}\;\left(V\right)=415.72-0.881A-0.293V_{c}+1050f_{z}+0.1\times 10^{-2}V_{c}^{2}+5417f_{z}^{2}+0.33\times 10^{-2}AV_{c}+6.25Af_{z}+V_{c}f_{z}$$

(17) $$\sigma_{ave}^{RS}\;\left(\mathrm{MPa}\right)=75.9+9.22A+0.013V_{c}^{2}+0.0375AV_{c}+182Af_{z}-31V_{c}f_{z}$$

According to the ANOVA results presented in Table 3, it is seen that the vibration amplitude for all the SIAs has greatest impact on determining their values. On the other hand, it is seen that the terms *fz*^2^ for *Ra*, *Rz* and *Rt*; *Vc*^2^, *fz*^2^, *A.fz* and *Vc.fz* for *Rsm*; and *Vc*, *fz* and *Vc*^2^ for *σ^RS^* are counted as insignificant terms since their p-value is greater than 0.05. Therefore, they can be eliminated from the corresponding SIA. Also, it is seen that all the terms are significant for determining the hardness. Moreover, the correlation coefficient factor *R*^2^, for all the modeled SIAs, is close to 100%, which implies that the model is accurate enough to navigate the design space.

### 3.2. Verification of Fatigue Life

**Figure 4:**
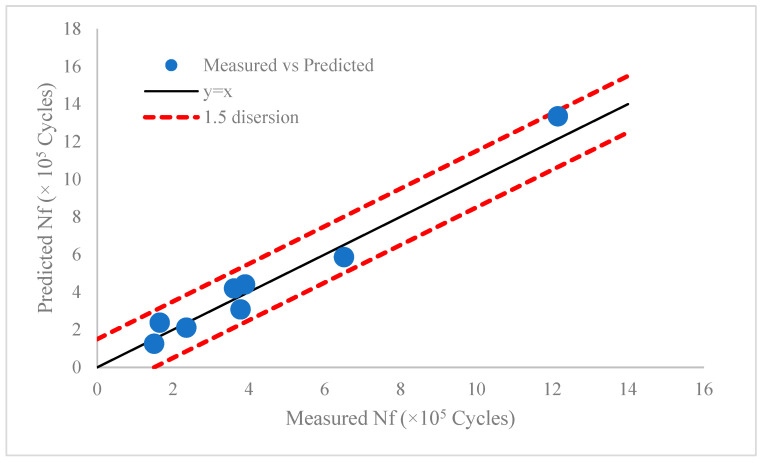
Comparison of measured and predicted values of fatigue lives for samples machined by different settings and under different loading conditions.

The fatigue life model was checked in two different loading conditions with maximum stress values of *σ_max_* = 750 MPa and *R_σ_* = 0.1 and *σ_max_* = 600 MPa and *R_σ_* = 0.1 to check the suitability of the proposed methodology under different loading conditions. For each loading condition, four series of samples under different machining settings were examined and the measured, predicted values of fatigue lives together with corresponding errors are presented in Table 4. Also, Figure 4 illustrates the distribution of experimental vs. predicted fatigue lives around the *y = x* line, which lies at 1.5 times the dispersion band. From the results presented in Table 4 and Figure 4, it is seen that there is close agreement between the measured and predicted values of fatigue lives. It is seen that for medium cycle fatigue where the stress amplitude is bigger than half of the yield stress of material, the absolute error values varies between 10 and 25%, while this value for high cycle fatigue is in the range of 9.88 to 18.52%. The error is within the acceptable range for fatigue life prediction in machining processes reported in the work of Song et al. [5].

Also, the prediction error can be compared with the work of Klotz et al. [12], where they a used micromechanical fatigue crack propagation model for the prediction of fatigue life in machining processes. It should be pointed out that, nevertheless, they achieved a prediction error of 2%; in their work, they only checked the model for a single specific machining condition and the robustness of their results is still unclear. Moreover, in their model, the fatigue life was corelated to the measured SIAs not to process factors. However, in our work, the fatigue life was studied under different processing conditions as well as at two loading amplitudes. In other words, thanks to using a hybrid framework for the modeling of fatigue, the fatigue lives are directly correlated to process parameters. Therefore, it opens the option to adjust the machining process factors for anti-fatigue purposes.

Moreover, it should be noted that based on previous research using a stress-based approach, another reason for the mismatch between the measured and predicted values of fatigue is that the approach is a micromechanical methodology that does not count the propagation of fatigue cracks within the microstructure of the material. However, it has more convenient and faster implementation compared to micromechanical approaches that can only guarantee better results in some cases but with a more complex implementation algorithm that takes more time to be implemented [13].

### 3.3. Parametric Influence

**Figure 5:**
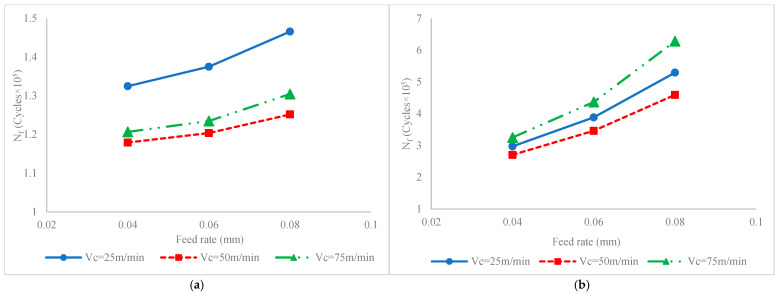
Interaction effect of feed rate and cutting velocity on fatigue life for (**a**) conventional milling and (**b**) ultrasonic assisted milling.

Once the model is verified by confirmatory experiments, it can be utilized to analyze the impact pf process factors on lifetime. The impact of process parameters on fatigue lives considering their interaction with cutting velocity and fatigue life can be seen in Figure 5. It should be noted that since the fatigue lives versus process factors are varied following the same trend under applied loads of 600 MPa and 750 MPa, only variation in one of them (*σ_max_* = 750 MPa) has been considered for analyzing the parametric influence.

**Figure 6:**
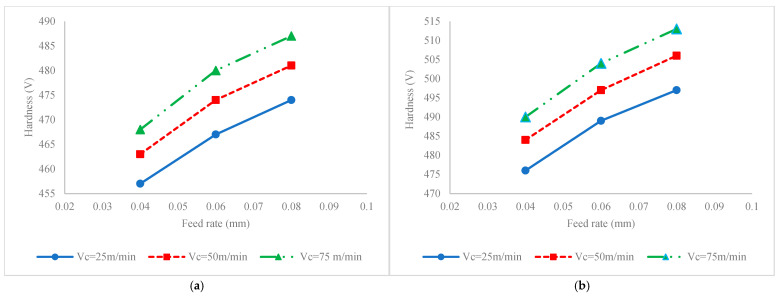
Interaction effect of feed rate and cutting velocity on mean hardness distribution for (**a**) conventional milling and (**b**) ultrasonic assisted milling.

**Figure 7:**
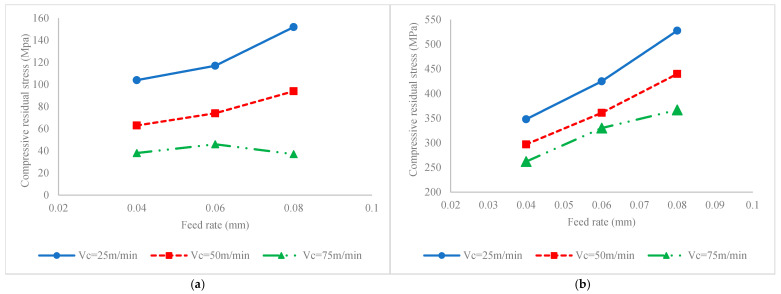
Interaction effect of feed rate and cutting velocity on mean compressive residual stress distribution for (**a**) conventional milling and (**b**) ultrasonic assisted milling.

**Figure 8:**
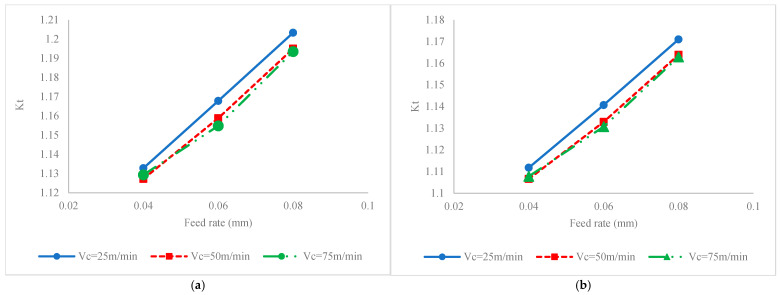
Interaction effect of feed rate and cutting velocity on SCF for (**a**) conventional milling and (**b**) ultrasonic assisted milling.

**Figure 9:**
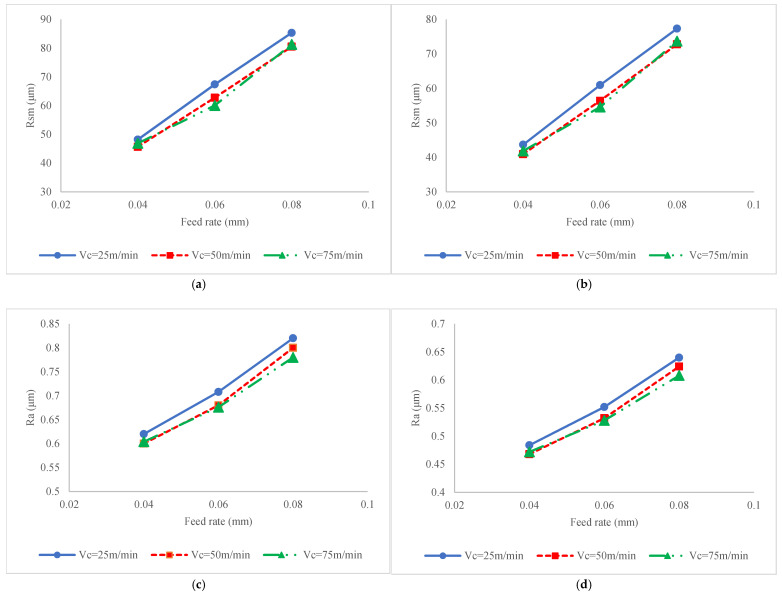
Interaction effect of feed rate and cutting velocity on (**a**) *Rsm* in conventional milling; (**b**) *Rsm* in ultrasonic assisted milling; (**c**) *Ra* in conventional milling; and (**d**) *Ra* in ultrasonic assisted milling.

Figure 5 illustrates the impact of process factors on fatigue life for conditions when the milling process is carried out without ultrasonic vibration (Figure 5a) or ultrasonic assisted milling (Figure 5b). To justify the variation in fatigue life, the variations in SIAs, i.e., hardness, residual stress, stress intensity factor and surface roughness, versus process factors are also presented in Figure 6, Figure 7, Figure 8 and Figure 9, respectively. It should be added that since the roughness attributes *Rt* and *Rz* are varied in a similar pattern to Ra, in order to optimize the figure space, only *Ra* is reported in the figures.

#### 3.3.1. Feed Rate

According to Figure 5, it is seen that, irrespective of the application of ultrasonic vibration and variations in cutting velocity, the fatigue life increases by increasing the feed rate. The justification can be found in Figure 6, Figure 7, Figure 8 and Figure 9. According to Figure 6, it is seen that increases in the feed rate result in increases in the hardness. Once the hardness increases, the fatigue limit stress, i.e., the stress required for the initiation of crack, increases. In other words, the resistance of material against fatigue cracks increases, which yields improvement in fatigue life.

Once the impact of hardness on fatigue life is identified, the physics behind the effect of feed rate on hardness should also be discussed to understand how the feed rate impacts the fatigue. The increase in feed rate results in a further cutting force that induces compressive load and higher temperatures on the work surface. The association of these two effects results in more thermomechanical strain to the material while it is softened because of further temperature increases that yield grain refinements and the formation of a work-hardened surface layer [14].

Also, as shown in Figure 7, it is seen that increases in the feed rate, irrespective of the existence of ultrasonic vibration and values of cutting velocity at 25 and 50 m/min, result in increases in the values of compressive residual stress. As stated above, this is due to further plastic deformation because of increases in the cutting force that result in more compressive load; in other words, the increased cutting temperature results in softening of the material and facilitates plastic deformation. Accordingly, the further the plastic deformation progresses while inducing compressive load, the greater the magnitude of compressive residual stress. It also needs to be added that the increased cutting temperature causes the formation of thermal stress that generates tensile residual stress. Specifically, when the cutting temperature is high due to further cutting velocity, the impact of the formation of tensile residual stress due to the high thermal load dominates the impact of generation of compressive residual stress due to facilitated plastic deformation (as a result of material softening at higher temperatures) and results in decreases in compressive residual stress when the feed rate and cutting velocity are set at their maximum values, i.e., 0.08 mm and 75 m/min, respectively.

Accordingly, the enhancement of fatigue life via an increased feed rate can also be attributed to enhancements in the magnitude of compressive residual stress. As a result of bigger magnitude of compressive residual stress, the fatigue stress reduces and results in further fatigue life [15].

Variation in the stress concentration factor is another fact that should be studied while analyzing the effects of machining parameters on fatigue. This factor, according to Equation (3), is mainly adjusted by surface roughness. According to Figure 8, it is seen that by increasing the feed rate, irrespective of the existence of vibration and values of cutting velocity, the SCF increases by increasing the feed rate. Accordingly, it has a negative impact on fatigue life because it intensifies the fatigue stress and results in a reduction in the lifetime. However, the range of variation in SCF by the feed rate is not that significant compared to the enhancement in values of hardness and residual stress and it cannot negatively impact the fatigue life.

The increase in SCF by the feed rate is due to variation in the surface roughness. Once the feed rate increases, the surface profile that is generated as result of the engagement of the tool nose and workpiece surface includes a bigger part of intersected circles (i.e., trace of the tool nose on the material surface) that results in the formation of profiles with deeper valleys, which increases *Ra*, *Rz* and *Rt*, as well as results in a wider uneven pitch, i.e., *Rsm* (as shown in Figure 9. Their association in Equation (3) results in a bigger SCF value. On other hand, it is seen from Figure 7 and Figure 8 that the feed rate is the parameter with the most significant impact on surface roughness and SCF compared to other parameters like cutting velocity and the existence of vibration.

#### 3.3.2. Cutting Velocity

According to Figure 5, it is seen that there is high interaction between the cutting velocity and vibration existence on fatigue life where, irrespective of the values of the feed rate, in the conventional milling process, the increase in cutting velocity results in reductions in fatigue life up to 50 m/min; then, through further increases in cutting velocity, the fatigue life slightly increases. On the other hand, in ultrasonic assisted milling processes, by increasing the cutting velocity up to 50 m/min, the fatigue life slightly decreases; then, by further increasing the cutting velocity reaching 75 m/min, the fatigue life is dramatically increased.

The impact of cutting velocity on fatigue lives can be justified by analyzing SIAs and SCF. According to Figure 6, it is seen that as a result of increases in cutting velocity, in both conventional and UAM processes, and irrespective of the values of the feed rate, the mean hardness distribution increases. Accordingly, the resistance stress against fatigue crack initiation and propagation increases and results in their postponement and declaration, respectively. Accordingly, the fatigue life increases. The impact of cutting velocity on hardness can be discussed as follows:

This can be attributed to the fact that by increasing the cutting velocity, as a result of the increased cutting temperature in the workpiece, material softening occurs that facilitates the process of plastic deformation and grain refining. In other words, the process acts as a thermomechanical sever plastic deformation (SPD) process in which the degree of plastic deformation and sequential work hardening is enhanced by increasing the cutting velocity [16].

The impact of the cutting velocity on residual stress can also be studied to underline its impact on fatigue life. As shown in Figure 7, it is seen that by increasing the cutting velocity, the magnitude of the mean distribution of compressive residual stress decreases. As stated above, the thermal load caused by higher cutting temperatures induced on the workpiece generates tensile residual stress on the surface and subsurface layers [17], which subsequently results in a reduction in compressive residual stress that intensifies the effect of fatigue stress.

The SCF induced by roughness indices is another factor that should be studied while analyzing the effect of cutting velocity on fatigue life. As shown in Figure 8, it is clear that by increasing the cutting velocity the SCF decreases. To explore this effect, the role of cutting velocity on roughness attributes, i.e., *Rsm* and *Ra*, should be identified. As shown in Figure 9, it is seen that by increasing the cutting velocity, the surface roughness decreases irrespective of the existence of ultrasonic vibration and values of feed rate. It can be stated that as a result of increased cutting velocity, because of higher temperatures, the probability of the formation of a built-up edge on the rake surface of the tool decreases and the formation of scratch and micro-notches, which are main source of stress intensity, are restricted. Owing to this fact, the SCF decreases, which results in limiting the impact of fatigue load on driving crack propagation.

According to the stated discussions, considering fatigue life as a machinability indicator, it is seen that increasing the cutting velocity has a positive impact on hardness and SCF and a deteriorating impact on compressive residual stress. However, these two positive effects dominate the negative impact and fatigue life is enhanced by increasing the cutting velocity.

#### 3.3.3. Ultrasonic Vibration

The impact of ultrasonic vibration on fatigue lives and corresponding SIAs and SCF can be compared based on the values on the vertical axes of Figure 5, Figure 6, Figure 7, Figure 8 and Figure 9. Accordingly, it is seen that as a result of the application of ultrasonic vibration, the fatigue life is also enhanced from 1.15 × 10^5^ to 1.45 × 10^5^ cycles and from 2.5 × 10^5^ to 6 × 10^5^ cycles. The impact of ultrasonic vibration on fatigue life can be justified by variations in SIAs and SCF.

According to Figure 6, it is seen that by applying ultrasonic vibration, the hardness range increases from the range of 547 to 485 V to the range of 575 to 510 V. This enhancement can be attributed to the addition of impact loading to the process. Therefore, the thermomechanical impacting process results in more plastic deformation and grain refinement that yields further hardness. Accordingly, as a result of the formation of hardened surface layers, the resistance stress against fatigue load increases and leads to enhanced fatigue life.

Also, it is seen from Figure 7 that by applying ultrasonic vibration to the process, the magnitude of mean compressive residual stress distribution increases from the range of 38 to 152 MPa to 262 to 528 MPa. As previously discussed, the synergetic effects of high frequency impacts and thermomechanical cutting results in the generation of work hardening of the surface layer and yields the generation of compressive residual stress on the surface of the workpiece.

The SCF induced by roughness is another attribute that is varied by imposing ultrasonic vibration on the milling process. According to Figure 8, it is seen that applying ultrasonic vibration causes a reduction in SCF from the range of 1.13 to 1.20 for conventional milling processes to 1.109 to 1.17 for ultrasonic assisted milling. This reduction in SCF is mainly attributed to the reduction in Ra in ultrasonic assisted milling compared to conventional processes, while the *Rsm* does not significantly change by applying the vibration. As a result of reciprocating motion between the tool rake face and workpiece, the sticking of workpieces on the tool surface is limited and results in a reduction in the probability of the formation of a built-up edge. Therefore, the surface roughness decreases by applying ultrasonic vibration [18].

Based on the foresaid discussion, it is said that the maximum fatigue life in the conventional milling process is obtained when the cutting velocity is 25 m/min and the feed rate is 0.08 mm, while in the ultrasonic assisted milling process, the maximum fatigue life is achieved by the selection of the same feed rate and cutting velocity, 75 m/min. This change in optimal settings can be attributed to the role of the adjustment of compressive residual stress versus cutting velocity in milling processes with and without ultrasonic vibration. As shown in Figure 7, when the process is carried out without ultrasonic vibration, the compressive residual stress decreases because of the generation of tensile thermal residual stress within the surface layers; however, by applying ultrasonic vibration as a result of high impact loads, the high cutting velocity facilitates the generation of compressive residual stress because of material softening and high-impact loading. In other words, it can be stated that the negative impact of a high cutting velocity in conventional milling processes on compressive residual stress and corresponding fatigue life can be changed to a positive impact when ultrasonic vibration is applied to the process.

According to the abovesaid discussion, it is inferred that applying ultrasonic vibration results in the enhancement of fatigue life by improving the residual stress state and SCF and by increasing the hardness. The former results in decreasing the amount of applied fatigue load, while the latter increases resistance stress against fatigue crack initiation and propagation. Accordingly, the fatigue life increases.

## 4. Conclusions

In the present work, a mechanistic model was developed to correlate the ultrasonic assisted milling parameters to fatigue life. Here, firstly, the vibration amplitude, cutting velocity and feed rate were correlated to surface roughness, residual stress and hardness using a second-order statistical regression model. Then, they were correlated to the fatigue life by using the mechanical stress approach. The obtained results can be summarized as follows:

- The model is able to predict the fatigue life of machined components for several machining conditions with an error range between 10.25% and 25.45%, while the maximum nominal fatigue load is 750 MPa and the error range of 9.88% to 18.82% when the four-point bending fatigue tests is carried out with the stress amplitude of 600 MPa. The error ranges lie between the acceptable values according to previous studies [5,6,13].
- It was found that the hardness alternation that corresponds to the fatigue resistance plays a predominant role in determining the impact of process factors on fatigue life, where the increase in machining parameters (cutting velocity and feed rate) as well as applying vibration results in increasing the hardness.
- The residual stress plays different roles with respect to the cutting velocity on fatigue life, while the increase in cutting velocity in conventional milling processes results in reductions in compressive residual stress and fatigue life; on the other hand, in ultrasonic assisted processes, the increase in cutting velocity results in further compressive residual stress and dramatically increases the fatigue.
- The stress concentration factor induced by roughness does not change significantly by process parameters compared to other SIAs. This means that the impact of roughness alternation on the determination of the fatigue life is not as significant as hardness alternation and residual stress distribution.
- The increase in the feed rate increases the hardness and compressive residual stress in each condition but, on the other hand, it increases the SCF. However, since the SCF effect on fatigue life is not as significant as hardness and residual stress, the fatigue life increases by increasing the feed rate.
- The application of ultrasonic vibration can significantly enhance the fatigue life up to four times compared to conventional milling processes thanks to the improvement of compressive residual stress and hardness as well as a reduction in roughness and corresponding stress concentration factors.

## Figures and Tables

**Table 1:** Mechanical properties of Inconel 718.

Yield Strength (MPa)	Ultimate Tensile Strength (MPa)	Young Modulus (GPa)	Hardness (V)
1360	1500	19.3	390

**Table 2:** Design matrix.

No.	Process Input					Surface Integrity			
	*A* (μm)	*V_c_* (m/min)	*f_z_* (mm/t)	*Rt* (μm)	*Ra* (μm)	*Rz* (μm)	*Rsm* (μm)	*σ^RS^ _ave_* (MPa)	*H_ave_* (V)
1	0	25	0.04	2.79	0.62	2.17	48.21	−104	457
2	0	25	0.06	3.186	0.708	2.478	67.4	−117	467
3	0	25	0.08	3.69	0.82	2.87	85.34	−152	474
4	0	50	0.04	2.7	0.6	2.1	45.69	−63	463
5	0	50	0.06	3.06	0.68	2.38	62.78	−74	474
6	0	50	0.08	3.6	0.8	2.8	80.55	−94	481
7	0	75	0.04	2.718	0.604	2.114	46.9	−38	468
8	0	75	0.06	3.042	0.676	2.366	60.05	−46	480
9	8	75	0.08	3.51	0.78	2.73	81.32	−37	487
10	8	25	0.04	2.18	0.484	1.694	43.68	−348	476
11	8	25	0.06	2.48	0.552	1.932	60.97	−425	489
12	8	25	0.08	2.88	0.64	2.24	77.35	−528	497
13	8	50	0.04	2.11	0.468	1.638	40.95	−297	484
14	8	50	0.06	2.39	0.532	1.862	56.42	−361	497
15	8	50	0.08	2.81	0.624	2.184	72.8	−440	506
16	8	75	0.04	2.12	0.472	1.652	41.86	−262	490
17	8	75	0.06	2.38	0.528	1.848	54.6	−330	504
18	8	75	0.08	2.74	0.608	2.128	73.71	−367	513

**Table 3:** ANOVA result for regression analysis of SIAs.

SIA	Statistic	Terms									
		*A*	*Vc*	*fz*	*A* ^2^	*Vc* ^2^	*fz* ^2^	*A.Vc*	*A.fz*	*Vc.fz*	*R* ^2^
*Ra*	*p*-value	0	0.015	0.083	0.005	0	0.163	0	0.003	0	99.93%
	Contribution (%)	51.22%	2.05%	45.11%	0.13%	0.33%	0.01%	0.55%	0.15%	52.61%	
*Rz*	*p*-value	0	0.017	0.081	0.008	0	0.191	0	0.02	0	99.11%
	Contribution (%)	50.91%	2.03%	47.66%	0.15%	0.35%	0.02%	0.61%	0.12%	50.91%	
*Rsm*	*p*-value	0.472	0.019	0.013	0.012	0.092	0.846	0.063	0.226	0.472	99.63%
	Contribution (%)	4.66%	1.34%	92.84%	0.39%	0.14%	0.00%	0.18%	0.07%	4.66%	
*Rt*	*p*-value	0	0.015	0.083	0.005	0	0.163	0	0.003	0	98.89%
	Contribution (%)	53.45%	1.02%	44.14%	0.10%	0.40%	0.02%	0.69%	0.12%	53.45%	
*σ^RS^*	*p*-value	0	0.167	0.228	0.075	0.627	0.01	0	0	0	99.87%
	Contribution (%)	85.68%	6.54%	4.75%	0.06%	0.00%	0.15%	2.27%	0.43%	85.68%	
*H_ave_*	*p*-value	0	0	0	0.001	0	0	0	0.001	0	99.98%
	Contribution (%)	56.45%	13.55%	29.02%	0.04%	0.45%	0.13%	0.29%	0.05%	56.45%	

**Table 4:** Comparison of measured and predicted values of fatigue lives for samples machined by different settings and under different loading conditions together with corresponding error percentages.

No.	Process Input			*N_f_* at *σ_max_* = 750 MPa			*N_f_* at *σ_max_* = 600 MPa		
	*A* (μm)	*V_c_* (m/min)	*f_z_* (mm/t)	*Measured* *(Cycles* ×10^5^)	*Predicted* *(Cycles* ×10^5^)	*Error* (%)	*Measured* *(Cycles* ×10^5^)	*Predicted* *(Cycles* ×10^5^)	*Error* (%)
1	0	25	0.04	1.65	2.0749	−25.45	3.9	4.4123	−13.08
2	0	25	0.06	1.5	1.2517	16.55	3.78	3.0865	18.52
3	0	25	0.08	2.35	2.1092	10.25	6.51	5.8615	9.98
4	0	50	0.04	3.61	4.1952	−16.21	12.15	13.3572	−9.88

## Data Availability

The data will be available upon reasonable request.
